# The Angina of the Throat

**Published:** 1874-05

**Authors:** A. S. Payne

**Affiliations:** Virginia


VOL. IV.—MAY, 1874.—NO. 5.

### Original Communications.

### THE ANGINA OF THE THROAT.

*QUINSEY.—EPIDEMIC TONSILLITIS A DISTINCT DISEASE.—THE DIFFERENCE BETWEEN TOXICOLOGICAL AND PATHOGENIC POISONS—THEIR RELATIONS TO EPIDEMIC DISEASES CONSIDERED. DIPHTHERIA—ITS PARASITIC ORIGIN.—THE THEORY OF OZONE CRYPTOGAMIA DISCUSSED.*

BY A. S. PAYNE, M.D., OF VIRGINIA.

It is a divine truth, that from the "fullness of the heart the mouth speaketh;" for here I sit down to prepare a paper on small-pox, and find myself running off into the angina of the throat.

To arrive at a correct diagnosis, there are few diseases requiring at the hands of the practitioner of medicine a more careful examination than these anginose diseases of the throat. More particularly is this the case when their visit is paid in an epidemic form. Hence it is we find epidemic tonsillitis, a distinct disease, confounded with common quinsey, with scarlatina, with the ulcerative variety of diphtheria, with aphthæ, with thrush, and frequently with measles. Indeed, I have heard it called, by some physicians, rosalia; by others it is spoken of as a hybrid, between scarlatina on the one hand, and measles on the other. The semeiology of common quinsey or sporadic tonsillitis and those of epidemic tonsillitis are widely distinct,—the one always sthenic in its character, the other invariably asthenic in its tendency. The symptoms of common quinsey are so plainly, so strongly marked, that they cannot possibly be mistaken for any other disease; the symptoms of epidemic tonsillitis so faintly drawn, that it may be easily mistaken for several other of the anginose diseases of the throat. It is a frequent visitor in mountainous regions of country, where the climatic alternations are sudden and frequent. Unlike most eruptive "epidemic diseases," one attack affords no immunity from a recurrence of the disease, but rather predisposes to subsequent attacks. Children with thin skins, and of a sanguineous temperament, as a general thing, are most severely affected.

There is an eruption that belongs to epidemic tonsillitis which is pathognomonic, when seen, but, unfortunately, it is frequently suppressed. The eruption, in appearance, is between the measles and scarlet fever,—too pointed for scarlet fever, and hardly enough so for measles. Although if you rub your hand over the eruption, it has a harder feeling, is more papulous in its character, yet it is not as highly pointed as is the case in measles. I have seen this eruption, on the lower extremities of patients, assume a black color, and bearing a striking resemblance to varioloid. There is often a soreness of the scalp, followed by the eruption appearing just beneath the hair, and passing downward, just as is seen in small-pox. But I have never seen it appear upon the soles of the feet; nor does it travel by systematic stages, as small-pox is known to do. The mouth and posterior fauces show aphthous ulcerations, and there is usually some slight swelling and external soreness of one or both tonsils. As convalescence becomes established, the epidermis will be found to "bran off," instead of coming off in large flakes, as it does in scarlet fever.

In peculiar idiosyncrasies belonging to the tegumentary tissue of some patients, this disease would take on the appearance of erysipelas. Losing its own identity, this disease may degenerate into putrid sore throat; or the ulcerative process may go on unnoticed in the nares and posterior fauces, and result in death.

This epidemic was prevalent and widespread in this portion of Piedmont, Virginia, in the fall of 1872. It commenced in the latter part of August, and continued up to the first of December.

During the months of October and November, it began to affect the horses, very few farms escaping its ravages. It was rare for a lying-in woman to entirely escape from this disease, and often their lives were seriously endangered. In most of the cases occurring in lying-in women, this disease was peculiarly prone to take on the livery of erysipelas, but there was always some symptom present by which we could identify the influence of the prevailing epidemic.

I think I hazard nothing in pronouncing the widespread "epizootic" of 1872 an epidemic tonsillitis, typhoid in its tendency. In the horse, it may with propriety be termed a distemper, with strongly-marked symptoms, typhoid in their character. In the human, it might be termed, with equal propriety, a widespread epidemic of influenza, with a preponderance of typhoid symptoms. I remember to have had the disease in 1840, under the cognomen of the "Tyler grippe." I am aware that many able medical men in the North considered the "epizootic" trachitis, laryngitis, etc.; but I have given the diseases of the lower animals, for a number of years, much study, and I am sure, by careful examination, they will concede, with me, that it was really an epidemic of tonsillitis, affecting, in some portions of the country, the human and the horse alike. Very few cases of sthenic tonsillitis, or quinsey, if seen early, will die under the steady administration of blue mass, followed each morning by sulphate magnesia, with a fly blister to the throat. If you are called too late, and matter has formed, scarify the tonsils, and let it out with as little delay as possible. In ordinary cases, I use the coal oil to the throat, to be well rubbed in; flannel to be worn around the neck. In the epidemic or asthenic variety of the disease, the system must be sustained by stimulating remedies and nourishing diet, to enable the "*vis medicatrix naturæ*" to rally her forces, and come to the rescue. The ulcerations should be touched once thoroughly, by a saturated solution nitratis argenti, and then followed by a gargle of chlorate of potash and syrup of quince seed. Of the internal sustaining remedies, the chlorate potassa, Labarraque's chloronated solution, chloride ferri, pulvis zinziber, and quinine, will be found the best.

But before I go further into the etiology, semeiology, etc., of these angina, I wish to quote what has been so ably said upon the subject of poisons by my friend, the late Dr. Dowler, of New Orleans:

"In strict propriety poisons belong exclusively to the domain of toxicology, and are not assignable to the pathogenic category. There can be no harm however, in so extending the signification of the term "poison" to include every agency known and unknown. What acting on the organism causes it to depart from the healthy standard, provided in so doing, we do not compound together objects which are entirely distinct, and draw parallel conclusions from the most divergent premises. Let us therefore follow the acologists so far as to give this extension to the meaning of the term; and then it will necessarily follow that poisons must fall into two great classes; namely: first the toxicological and second the pathogenic. Let us compare or rather contrast these classes of poisons, and determine how they may be identified with each other.

1. Toxicological poisons are cognizable, appreciable, tangible and material. Pathogenic poisons have hitherto proven in their external and material character, either unknown, unappreciable and intangible, as in their relations to cholera, yellow fever, etc., or known and appreciable only when incorporated in, and coexistent with animal matter as in syphilis, variola, etc., otherwise being wholly unknown.

2. The organic manifestations which attend the action of toxicological poisons are remarkaby invariable and uniform, and respond to the agent administered. The organic manifestations, in those who have been exposed to the action of pathogenic poisons, are so variable in intensity that they may be either absolutely null, or exist in every degree, from the modest form of the disease to the most deadly, as is exemplified in cholera, yellow fever, small-pox, etc.

3. With toxicological poisons, the uniformity of the effect and organic susceptibility are remarkable. With the pathogenic poisons of yellow fever, cholera, diphtheria, etc., the want of this organic uniformity in susceptability is remarkable, absolute immunity attending one person, whilst a second is lightly, a third severely, and a fourth mortally attacked in the same appartment—proving first, that the pathogenic or objective cause must necessarily be endowed with an uniform potency; and secondly, that the variability of the organic phenomina must be owing wholly to unknown internal susceptabilities, and not owing to variable intensities of the prthogenic agency.

4. The intensities of toxicological poisons are known, determinate demonstrable. The intensities of power in pathogenic poisons we have inferréd to be uniform as in cholera, yellow fever, diphtheria, etc. This does not however enable us to determine the measure of intensity of that power, as characterizing it under the denominations of strong or weak. For example, immunity from the disease might be urged to prove the intensity of organic resistance to a powerful pathogenic agent, whilst resulting mortality might be urged to prove intense organic susceptability to an extremely feeble agent.

5. Organic toxicological poisons are normal, and do not reproduce themselves in the organism, in which they induce toxicosis, as the poison of crotalus, scorpion, hornet, etc. Pathogenic poisons derived from an organic source are abnormal and tend to reproduce themselves, as the poison of syphilis, glanders, hydrophobia, etc.

6. Toxicological poisons act with increased energy and danger, on the young, the debilitated, the infirm, etc.; females being more easily affected than males. The pathogenic poisons of yellow fever, cholera, etc., as manifested in the same apartment, often destroy the powerful and robust, while the tender infant and the invalid are lightly affected.

7. The treatment of toxicological poisoning is strictly antidotal, and with reference to throwing off, counteracting, rendering inert, and neutralizing the poisonous agent. The treatment of pathogenic poisoning as in yellow fever, cholera, etc., is strictly antipathic, and is addresed to the morbid conditions present, and not to any assignable cause of those conditions.

8. No known toxicological poison is capable of producing organic conditions and manifestations, identical with those exhibited in pestilential diseases. No pathogenic poison is capable of producing organic conditions and manifestations identical with those superinduced by toxicological poisons.

8. Toxicological poisons generally teach avoidance by appealing to our sight, hearing, taste, smell and touch. Pathogenic poisons as in cholera, yellow fever, etc., have never yet objectively appealed to our external senses, nor do they give us notice of material existance. They are inodorous, invisible, intangible, inaudable, tasteless.

10. Toxicological poisons administered in the maximum act with mortal force and without considerable lapse of time. Rabies canina forms an exception to this rule. Infants may be exposed to the continuous action of the pathogenic poisons of cholera, yellow fever, diphtheria, etc., for weeks and finally suffer a slight attack, whilst the most powerful and robust subjects may fall victims after a single day's exposure in the same apartment.

11. Feeble toxicological poison produce feeble effects on the powerful physical organism, and it is not possible for them, generally to cat otherwise. It is not at all improbable that the obscure pathogenic poisons of yellow fever for instance, may be intrinsically an extremely feeble agent, acting on variable susceptabilities. The same may apply to the pathogenic poison of cholera, determining its violence, rendering small-pox distinct or confluent in the same apartment and in the same manner determining the intensity of scarlet fever as simple angenose, or malignant.

12. The existance of pathogenic poison is merely inferential, they having no sensible properties, nor have we, apart from animal matter, any conception of the material cause of variola, rubeola, equinea, or syphilis, no such substance as variolon, rubeolon, equinon, or syphilon ever having been either discerned, or heard of. Dr. Mitchell claims to have discovered septon it is true, but little has been heard of it since his time. The existence of certain external entities determining yellow fever, cholera, etc., is an *ex itæcessato rei* and the only circumstance, which renders such conclusion inevitable is the mere ultimate, naked and inexplicable fact of the disease prevailing in one locality and not in another, declaring a local cause.

13. Toxicological poisons exhibits curative and therapeutical, as well as morbid effects, are employed as antidotes to each other, and act any number of times on the same subject. Pathogenic poisons are purely morbid, and are without therapeutic power. They merely as in small-pox, yellow fever, etc., confer immunity against a second attack.

The data on which it has been attempted to explain the origin of pestilential diseases, by the condition of the atmosphere in relation to ozone, are at once simply inadmisable. As a scientific achievement the disclosures in relation to ozone are most curious and important, but the etiology of epidemics has not advanced a single step thereby. Later investigations appear to have given a very satisfactory explanation of the nature of ozone, showing beyond a doubt that it is merely oxygen gas rendered chemically active and corrosive like chlorine, bromine, etc., by the action of electricity, and by this means from being inodorous and unirritating it acquires a strong odor and chemical activity, like these elementary bodies. Nothing, however, is known of this electrized oxygen on the animal economy, save that which relates to it as a toxicological agent. Sconben and others having shown that it is highly oppressive to the lungs, and that small animals are speedily killed by it. The pathogenic power of either the hyper-ozonic or an-ozonic states of the atmosphere in the production of epidemics, comes to us with no other reason or recommendation than the appearance of novelty, for the electrical etiology is not new. It is said that certain coincidences have been observed to exist between the existance of epidemics and the showings of ozone-scope. The whole circle of reputed causes can boast of equally strong coincidences, and by same system of reasoning, the whole might be proven to be true, at the same time, however contradictory these reported causes might be. A hundred contradictory causes, doubtless remain to be discovered, with equally invariable coincidences. The invalidity of the ozonic theory may be ligitimately inferred, without our going out of the chemical labaratory. The azone of natures labratory cannot differ from that observed by the chemist. An abnormal state of the atmospheric oxygen must necessarily be visited alike on all; and were such a thing possible as a dangerous hyper, azonisis of the atmosphere, the result would be necessarily an universal poisoning effect, as veritably toxicological as could be produced by chlorine gas. Whatever evils on the other hand might be produced by the an-azonic state must be equally uniform and toxicological. The strong would not be destroyed, and the weak spared, as is often seen in epidemics. The contrary of these positions can never be taken for granted. It must be proven. Nothing but the strongest and most positive proof can ever be admitted to establish the ozonic explanation of epidemic diseases. The pathogenic poisons of cholera, yellow fever, etc., are endured with habitudes, which wholly differ from anything which has ever been discovered, and by nothing which has been discovered can these diseases be excited so far as is known. "Nothing but direct experimental proof can be admitted. The pathogenic power can never be admitted on a weaker proof than the toxicological. Both require demonstration and the former is disproven by the latter."

Whilst influenza may be defined to be an "epidemic catarrh," yet it is not improper to speak of an "epidemic influenza," because influenza may prevail in an epidemic, endemic, or sporadic form. In the epidemic form, it is usually more asthenic in its character than in either its endemic or sporadic form. I believe the tonsils to be the tissue usually found primarily affecteed; but I do not deny that laryngitis, trachitis, and even pharyngitis, may follow quickly and closely on the idiopathic or primary tonsillitis.

Several eminent German writers consider diphtheria to be of parasitic origin, and one powerful advocate of the cryptogamic origin is found in the late Professor I. K. Mitchell, of Philadelphia. The conclusions of Professor Hallier we do not accept, for three reasons: First, because the fluids sent to him were not put up with proper precaution for the exclusion of extraneous spores; second, because the culture apparatus used by him does not give reliable results, as we have found by experiment; and lastly, because his reasoning is based upon a peculiar theory of his own, that parencellium, mucus, etc., are merely unripe forms of certain cartilaginous fungi—a theory that cannot be discussed here, but of which it is sufficient to say that it has been accepted by no other mycologist. The statement of Dr. Stiles, that the fungous origin of all zymotic diseases is now conceded by the highest authorities in mycological research, will, no doubt, surprise the said authorities; for Berkley, Curtis, and DeBarry, the highest authorities in England, America, and Germany, most assuredly concede nothing of the kind. In reference to the parasitic origin of epidemic diseases, Dr. Leidy truly says: "In many animals, entozoa and entophyta are almost never absent, and probably, when in their natural habitation, and few in number, or not of excessive size, are harmless."

Many important diseases have been supposed to originate from parasitic animals and vegetables. The former are not the true entozoa, for they are too large, and may be detected by the naked eye; but they are considered to be animalculæ so small that they cannot be discerned even by the highest powers of the microscope. But, independent of the fact that the existence of such entities is a mere suspicion, none of the well-known animalculæ are poisonous.

At various times I have purposely swallowed large draughts of water containing myriads of monas, vibrio enzlenia, volcox leucophyn, votacella, etc., without ever having perceived any subsequent effect. The production of certain diseases, however, through the agency of entophyta, is no longer a subject of doubt, as in the case of the muscardeum in the silk-worm, mycodem of prurigo-favosa in man, etc.; but that malarial and epidemic diseases have their origin in cryptogamic vegetable or spores, requires a yet simple proof. If such were the case, minute vegetables and spores, conveyed through the air, and introduced into the body by respiration, could be detected. The minutest of all known living things is the vibrio leneola of Moller, measuring only the 36,000 of an inch, and the smallest known vegetable spore is very much larger than this, whilst particles of inorganic matter can be distinguished the 200,000 of an inch in size. I have frequently examined the rains and dews of localities in which intermittents were epidemic, upon the Schuylkill and Susquehannah rivers, but without being able to detect animalculæ, spores, or even any solid particles whatever. I have examined the air itself for such bodies, by passing a current through clear water. Ordinarily, when the atmosphere was still, early in the morning or in the evening, neither spores nor animalculæ could be detected. When piles of decaying sticks or dry leaves were stirred up, or the dust was blown about by the wind, a host of most incongruous objects would be obtained from the air—none, however, which could be supposed capable of producing disease.

To assent, under these circumstances, that there are spores and animalculæ capable of giving rise to epidemics, such as diphtheria, but not discernible by any means at our command, is absurd, as it is only saying, in other words, that such spores and animalculæ are liquid, and dissolved in the air, or in a condition of chemical solution. That the air may be poisoned by matter incapable of detection by the chemist, is proven by the emanations from plants, as the rhus senem hippomane, maricella, etc.

The violence of diphtheria appears to be equally aggravated by domestic uncleanliness, certain predisposing individual conditions, and want of hygienic arrangements. In other words, it owes its origin and successful propagation to the same laws we have found governing all other epidemic diseases, requiring an exciting and a predisposing cause, as we find to be the case in typhus and typhoid fevers, to reproduce itself. Its origin is the result of a pathogenic poison, invisible, intangible, and as yet unknown.

The following, from the pen of Dr. Henry Hartshorne, is so valuable that, if he had never written another word upon medical science, it is of itself sufficient to immortalize his name:

"*Mode of Propagation.*—The leading facts in the etiological history of diphtheria are that it is usually epidemic, and that its visitations are remarkably limited, 'acting with intensity in confined centres'—as a small village, a crowded school, a numerous family, often inflicting thereon a terrible loss, as a sort of domestic panic and pestilence.

"The name diphtheria is derived from the Greek word *διφθερα*, a prepared skin or hide. It is well understood to be a modification sanctioned, though not suggested, by Dr. Farr, the English registrar general of diphtheritis, which was introduced by Bretonneau, of Tours, in 1826. Other appellations, by which the same affection has been at different times and places recognized, are: Ulcus Egypticum, angina maligna, angina pellicularis, angina cum tumore, glossitis, putrid sore throat, pseudo-membranous pharyngitis, etc.

"Very little allusion was made to this disease in medical works or journals, especially in America, within the twenty-five years preceding the last four or five. Some may have even supposed that a term so much in use at present, so rarely before, indicated the advent of a new disease; but a little inquiry shows that is far from being the case. I find that quite a list of distinguished authors may be quoted who have described pseudo-membranous inflammation of the fauces and pharynx, with or without laryngeal extension, and this is the definition of diphtheria. The earliest of these authors was Aractus, of Cappadocia--probably a cotemporary of the Emperor Vespasian—who describes, as an Egyptian and Syrian epidemic, an ulcerative affection of the tonsils, in which they are covered with a white, livid, or black crust, and in which death mostly took place by asphyxia, preceded by loss of voice. The only other ancient authors who appear to have distinctly alluded to it are Macrobius and Cachus Aurelian.

"In more modern times, Canevola, at Naples, in 1620, and Ghisi, of Cremora, in 1740, gave accounts of its epidemic prevalence, the latter being the first to discern the pseudo-membranous nature of the deposit peculiar to the disease. In Spain, amongst others, Lomazo, of Madrid, described it in 1622, giving it the name of *garotillo.* In France, it was discussed by Chomel, in 1749; by Gueinser, in 1821; and by Bretonneau, in 1825—the name of the latter having been since that time especially identified with it. The earliest English writers who are quoted as alluding to it are Huxham, in 1757, Fittergill, about the same period, and Cheyne, who distinguished it from croup, 1801. To these we may add Rosen, in Sweden, 1761; Albert and others, in Germany; and in this country, Samuel Baird, of New York, 1761, and Stephen Brown, 1828.

"Ot the more recent medical writers who have dealt with it, the principal ones are Roche, Valleux, Grissolle, Rilliot and Barthez, Trousseau, Isambert, Bouchent, Empis, and Begnorel, in France; Frederick Rokitansky and Virchow, in Germany; Wolson, Hallam Greenhow, Wade, Harley, Laycock, Rogers, Simson, Bristowe, Hart, and Saunderson, in England."

In this country, the malady has been a less frequent visitant. I have had but a poor chance to ascertain what place it has in our recent medical literature, but mention must be made of Hustin Hunt and Jacobi, of New York, and the able paper of Dr. Geddings, of Charleston, S. C.; of Professor John P. Harrison, of Mississippi; and of the account given by Dr. Fourgeand, of Sacramento, of a severe epidemic of diphtheria in California, in 1856–7. Drs. Wood, Condie, and J. F. Meigs, of Philadelphia, and Slade, of New Jersey, give, in their respective treatises, a good account of the disease. I must not forget to mention the valuable experiments of Dr. Tucker, of Baltimore, who fell a martyr to the disease whilst engaged in conducting his interesting experiments. To Dr. James B. McCaw, Thomas Pollard, and the late David H. Tucker, of Richmond, Virginia, we are indebted for valuable monographs on diphtheria. We have from the pen of Charles Minor, M.D., University of Virginia, a most valuable paper on diphtheria. I must name J. W. Claiborne, J. Herbert Claiborne, J. H. Duval, of Petersburg, and R. W. Paxon, of Dinwiddie, as gentlemen who have contributed valuable papers on diphtheria. I have no doubt there are other gentlemen, in my own and neighboring States, who have likewise contributed to the history of this disease, whose papers are alike creditable to themselves and to their mother States; but, unfortunately for me, I could not place my hands on their recorded articles during the preparation of this paper.

The principal epidemics of diphtheria have been as follows: In Rome (described by Maccobius), A. D. 380; in Holland, 1337; at Paris, 1376; Naples, 1618–19; in Spain, 1620–22; in Sweden, 1761; in France, in 1810. In America, its earliest prevalence was in New York. It has been, since 1818, often localized in France, Spain, and Germany, and occasionally in England. The most important late epidemics of it have been those of Paris and Boulogne, in 1855–7, and its migratory invasion of England, passing from the southeast to the northwest. In this country, only a few cases attracted attention in 1857–8. We have a direct account of its having prevailed extensively in New York, Albany, Newark, Iowa City, Lynchburg, Waynesburg, and very fatally in Highland county, Virginia. Since 1857–8, I may say it has ravaged the whole Piedmont region of Virginia.

*Semeiology.*—The premonitory symptoms of diphtheria are much the same in its different forms, only varying in degrees and duration. They are languor, malaise, anorexia, swelling of the lymphatic glands behind the jaw, and slight sore throat. For convenience of description, we may classify the cases as they occur, into simple, croupous, ulcerative, and malignant diphtheria. All of these forms are more frequent and fatal in children than in adults. The simple variety is at once the mildest and most common of all the epidemics recorded. Its symptoms, described in brief, are fever, headache, furred tongue, constipation, dysphagia, with swollen and red or purplish tonsils, palate and fauces; upon one, or sometimes both, of the tonsils, often as early as the second or third day of the attack, a white or yellowish-white pseudo-membranous deposit being seen. The deposit softens and falls off spontaneously in a few days, or it may be removed by local appliances, sometimes forming again. Its removal leaves no ulceration or abrasion of surface behind it. The duration of the whole attack is from five to nine days, and the prognosis is favorable.

The croupous form is that which has proved so fatal in England and in our own country, and, so far as we can judge, elsewhere. I

should estimate nine-tenths of those cases terminating fatally to be of the croupous variety. It is especially frequent in children—most of all after measles and scarlatina—following, when idiopathic, the same prodromata as those observed in the simple variety, although generally more violent. From the beginning, a severe dysphagia comes on, with a yellowish-white, leathery exudation over the tonsils and fauces, the tissues of which, under or around the deposit, are reddened or swollen. The entrance of the disease to the larynx, which may often take place quite early, is marked by the usual symptoms of croup, in the cough, voice, and breathing, which it were needless to describe here. A fatal result takes place, in very many cases, in from two to five or six days, by asphyxia, although it is not impossible for recovery to occur by the expulsion of the membrane.

The ulcerative form is the one which (when destructive ulceration of the palate and tonsils has occurred, with dark-greenish colored and pulpy exudation, and some extravasation of blood, perhaps actual hemorrhage) has sometimes been mistaken for gangrene, and may have thus given rise to some of the familiar descriptions of gangrenous or putrid sore throat. At the same time, the occasional occurrence of true gangrene cannot be denied. M. Basquerol has given an account of an epidemic at the "Hospital des Enfans Maladies," in Paris, in 1841, in which many such cases occurred. The term septic diphtheria is applied by some writers to this type of the disease. The ulcerative is perhaps less fatal than the croupous variety, but it may undoubtedly destroy life, or, failing in this, leave the structure of the palate considerably impaired. Huxham described, in 1757, an epidemic of "ulcerous sore throat," occurring in Cornwall; and curiously enough, several towns in Cornwall, in later years, have been the seat of similar ulcerative epidemics, described by Dr. Burdon Sanderson, in an essay on diphtheria.

The malignant type is attended, at its commencement, by extreme headache, sometimes by vomiting (which is uncommon in the milder varieties), and by hemorrhage from the nose, rectum, mouth, etc. Great dysphagia occurs, and enormous engorgement of the submaxillary, parotid and cervical glands. The tonsils, pharynx and palate are covered thickly with a yellow or brownish, leathery deposit, soon becoming of an offensive odor. The tonsils may suppurate, or even slough. The nostrils are sometimes also involved. Extreme adynamia comes on at an early period, with a very rapid pulse, livid pallor of the face, and at first morbid heat, followed by clammy coldness of the skin, and coma often precedes death. Such cases may terminate fatally in three, four or five days, occasionally in *one* or *two*, sometimes, by the potency of the constitutional impressions, before the local affection has been fully developed.

Besides the symptoms which have been thus briefly enumerated, albuminnria is generally present in severe, and often in mild, cases of either form of diphtheria. This was first found out by Mr. Wade, of Birmingham, England. It occurs early in the attack,—is *not*, as has been shown by Dr. Burdon Saunderson, attended by any deficiency of the excretion of urea, and is, therefore, no indication of the dependence of comotose symptoms, which sometimes occur upon the state of the kidneys.

One more variety of diphtheria must be mentioned, which is less common than either of those which have just been described. It is the cutaneous—*i. e.*, pseudo-membranous inflammation of one or more parts of the integuments connected especially with wounds, etc. This has been observed repeatedly in France, having been described by Trousseau in 1830; by Robin in 1847. The sequelae of diphtheria has been especially studied by Drs. Fause, Moevyamt, Bonelton, LaGrange, Grill and Kinssford. They are great and long combined debility, loathing of food, paralysis of the soft palate, and general paralysis. In the latter, vision, deglutition and articulation, as well as locomotion may be involved. Death sometimes follows in a few weeks, but in a number of instances, recovery has occurred, etc., from three to eight months.

*Morbid Anatomy.*—The pellicular or pseudo-membranous, concretion, found upon a highly injected and tumified surface, or degenerate ulcerative, constitutes the anatomical peculiarity of the disease. This pseudo-membrane has been shown to be a coagulation of fibrin. A remarkable observation of M. Empris, of Paris, proved its independence after exudation of the tissues upon which it is thrown out—viz., that it concreted repeatedly and distinctly, within a tube left in the larynx after tracheotomy. Examined minutely the pellicle or membrane is found to vary from one-twentieth to one-eight of an inch in thickness, and to be fibro-laminated, *i. e.*, consisting of layers of net-work, including epithelial cells, and exhibiting on its cell surface, exudation corpuscles, or pyoid, globules and granules, which forms appear to be only stages of degeneration. No process of developement or organization occurs on the mass, it is aplastic. In some cases only a granular, superficial infiltration of the epithelium is observed. The ordinary deposit of diphtheria differs from the membrane of sporadic or idiopathic croup, and still more from the coaguable lymph of inflamed serous surfaces in being thicker, more tough, more yellow and less capable of organization. Dr. Burdon Saunderson, however, found evidences of developement of the exudation in one or two specimens of the simple type of diphtheria. Bretonneau, and afterward Dr. Saunderson, imitated the diphtheritic exudation, by injecting an oleaginous preparation of cantharis into the throats of animals. The only observable difference between the two exudations was the absence, in the diphtheritic, of a tendency to organization, which was evident in the cantharidal pseudomembrane. There would appear, in some of the cases of diphtheria, especially in those described by the German writers, an infiltration of the tissue of the mucous membrane itself; while in other instances a corrosive property secures to the exudation, causing a loss of substance in the parts in which it rests. This is especially dwelt upon by Rokitansky. In this connection, I will mention that, in 1852, I had the honor of presenting to the old Medical Society of Virginia a most beautiful, and perhaps perfect, specimen of "tubular false membrane." It was greatly admired by the members of the American Medical Association, none of whom had ever seen anything of the kind the equal of this specimen. In the July number of the *Stethescope* for the year 1852, you will find a record of the case from which this specimen was obtained by me. The intensity of the inflammatory process would hardly seem alone capable of accounting for the character of the diphtheritic exudation. It is more probable they are owing to a modification of the blastema, dependent upon the general state of the blood—a view suggested of the phrase used in the first edition of Rokitansky's great work, "filumous crasis." The second and third varieties of croupous exudation of that work compared very nearly with the description of the diphtheritic deposit given by various observers. In one particular, however, a difference is notable. The German pathologist mentions as one characteristic of all "croupous exudations," the often slight vascularity of the diseased texture. In diphtheria, the tissue of the fauces, tonsils, and pharynx, is always highly vascular.

The comparative history of these exudations oblige us to recognize in them an exception to the general rule of Paget, that the proportion of fibrin in an inflammatory lymph, and the fewer its corpuscles, the greater the probability of its organization, and, of course, the less of its degeneration. The diphtheritic exudation is especially fibrinous, and characteristically prone to degeneration. We are led thus to lean more to the view of Rokitansky, which dwells with greater emphasis upon the quality of the fibrin itself, as dependent upon the state of the blood, and to bear in mind the importance of the circumstances which determine, in the corpuscles of lymph, whether they shall undergo development or degenerative change.

In regard to the pathology of diphtheria (as names are arbitrary while things persist), we must ask, first, whether we are agreed to include under the term all affections in which a more or less tenacious concretion is observed in the mouth or throat—such as croup, aphthous inflammation, and thrush, or muguet; or whether we shall reserve the name diphtheria for a certain special disorder, usually epidemic or endemic, and differing pathologically and chemically from either of the diseases to which the above names are commonly applied. Notwithstanding the confusion which has existed in the works, especially of the early French writers, upon the subject, I believe the time has fully come for adopting the latter of these alternatives. I believe diphtheria to be a special toxicæmia or dyscrasia, with a peculiar inflammation as its local manifestation, having analogy in this respect to erysipelas, puerperal fever, the exanthemata, etc.; and, in illustrating the proposition common to many writers, but very well expressed by Paget, that each morbid condition is prone both to produce an inflammation in a certain part, and to give to that inflammation a certain form or character. Basing our methods of diagnosis upon the same principles, we must hold that diphtheria is to be distinguished from scarlatina, from croup, from thrush, and from aphthous inflammation of the fauces and throat. Much debate has occurred in regard to its identity or nonidentity with scarlet fever. Without going into an argument, it may suffice to quote the testimony of a late writer in the *British and Foreign Medico-Chirurgical Review,* that notwithstanding the fact that the diseases are often associated in a remarkable manner, abundant evidence has now been collected in England, that scarlatina is as distinct from diphtheria as from measles or small-pox. Diphtheria has prevailed when scarlatina had not been known for years, and mild epidemics of the one have occurred in coincidence with fatal epidemics of the other. In these early symptoms, more-over, there is a clear distinction in the eruption of scarlatina and in the peculiar punctated or brick-dust like flush of the throat and fauces, and strawberry tongue of that disease. The fact that, instead of preventing it, an attack of scarlet fever, as well as measles, predisposes to diphtheria, is established, and important.

From idiopathic inflammatory croup, as described by most writers since Horne and Cheyne—*i. e.*, that pseudo-membranous, laryngeal trachitis—we distinguish diphtheria by the following traits: Inflammatory croup is a sporadic and sthenic phlegmasia, in which constitutional symptoms are dependent as any inflammation upon the local affection; diphtheria is a *constitutional* disease, usually epidemic or endemic, in which the local symptoms are secondary to the general disorder. More directly in practice, we mark the difference between the two, in the commencement of the pseudo-membranous inflammation, in the region of the tonsils and pharynx, in diphtheria, instead of the trachea or larynx, as in croup, and in the fact that generally, when it invades the air-passages, the exudation does not, in diphtheria, extend to the trachea, but is confined to the larynx. At the same time, the symptoms resulting from such an extension are exactly those for which the designation croupal, or croupous, has been found the most convenient. Vogel asserted that the oidium albicani of muguet was to be found in the pellicle of diphtheria; but Dr. Laycock, Harley, Rogers, and others, have shown that the occurrence of this parasite, or of the lipthonia vuccalis, is, in diphtheria, merely accidental, and far from general. Diphtheria may, moreover, be distinguished from thrush by the commencement of the latter in the mouth, by the patches of exudation in diphtheria being much larger and thicker, and by the greater severity, usually, of the constitutional symptoms attending the diphtheritic attack. Thrush is also much less common in adults than diphtheria, and it is not epidemic. Roche and even Bretonneau have evidently confounded aphthous inflammation with diphtheria. The former of these writers states that the peculiar exudation of diphtheritis (angina conenneuse) is always at first vesicular. By universal consent at present, this character, with the smallness of the resulting ulcerations and their occurrence chiefly in the mouth, and in very young children, suffices to separate aphthæ from diphtheria.

Is diphtheria contagious? In my opinion, if you mean to signify by the term contagious a disease that transmits its own disease from one subject to another by direct contact without the assistance of any susceptibility or predisposing cause on the part of the patient, I should then say it was not contagious, and that very few epidemic or endemic diseases were so, strictly speaking. But if you mean by "contagious" to signify a disease from which exhalations or emanations may arise during its progress, capable of exciting a similar disease in those exposed to the influence of these noxious exhalations, or rather these deoxygenizing emanations, then I will say diphtheria is contagious, and most of these, epidemic and endemic, are more or less contagious, only requiring for their successful propagation that a susceptibility or predisposition should be furnished by the system. Diphtheria being the product of a pathogenic poison generated by climatic alternations or atmospheric distemperature. But in place of my own opinions, I will proceed to give you the different and conflicting authorities of this interesting subject: Guessert, Bretonneau, Vallein, Trousseau, Barthey, Bard and others have attempted to prove that it is contagious, whilst many others think it atmospheric. Dr Condie, Flint and others, have not been able to make up their minds upon this subject. The facts the contagionists appeal to are such as these: Two physicians, M. Herpen and Gendron, were attacked by the disease immediately after receiving on their faces a portion of diphtheritic matter coughed up by their patients. A soldier at Avignon using the teaspoon of a diphtheritic patient had buceal diphtheria. A boy in one of the French hospitals treading barefoot upon the spittal of a patient suffering with the disease, had psudo-membranous inflammation of the foot. On the other hand, Trousseau failed entirely to inoculate himself, and some of his pupils with the pseudo-membranous matter, and Harley's experiments with animals were equally without results. So far as my opinion is concerned, these experiments prove nothing. In my opinion, to successfully transmit disease from one to another by inoculation, it requires that the matter should be capable of undergoing in the system some kind of fermentation, or be injected into a vein, or thrown into the system with some force. I am lead to the above conclusion from the fact that I kept a cage full of rattle-snakes for six years in my office; inoculated dogs, cats and fowls, frequently and carefully with the virus; indeed, becoming emboldened by repeated failure, I inserted a little of the poisonous virus in my own arm, without ever having succeeded in producing any effect whatever. But let the snake itself bite the same animals or fowls, and you would quickly see its poisonous effect. The hypodermic method might produce a different result. The self-determining manner of migration of the disease moreover presents difficulties analagous to those connected with the history of cholera in the way of the theory of personal transmission, so that even those who insist most strongly upon its contagiousness are obliged to admit that its movements and invasions are by no means dependent upon such conveyance from individual to individual. It presents, in fact, a problem of great conplication to the epidemiologists. The following graphic account upon the subject of its migrations is from the polished pen of Mr. Earnest Hart, of London. "It visited," said he, "the open hamlets of the rural districts, and the crowded courts of the great cities; it reached the seaside, and fell with violence upon the infant population of Boulogne; it raged alike under the intense heat of Summer and during the cold Winter months; its ravages affected equally the inhabitants of marshy, ill-drained lands, and those of dry and elevated positions. Brighton has not escaped; Leasborough has suffered. It has swept across the marshy lowlands of Essex, and the bleak moors of Yorkshire; it has traversed the flowery lanes of Devon, and the wild flats of Cornwall that are swept by the sea-breeze. It has seated itself upon the banks of the Thames; scaled the romantic heights of the North Wales, and has descended into the cornish mines." The only cosmic influences which appear to exhibit any promotive agency in the development, are excessive alternations of temperature, and of the barometic state of the atmosphere. Like other zymotic diseases, it attacks with the greatest malignancy those places in which the public, and especially domestic hygiene are neglected or imperfect. The poor are therefore the greastest sufferers. But it is by no means restricted to them or to their dwellings. The effeminate influences of luxury and indolence unite it also to the homes of the affluent, where debility of constitution appears to supply the place of a fowl atmosphere, as a predisposing cause. The question which especially remains for solution is whether there exists for diphtheria a special morbific poison (as is the case of small-pox, autumnal fever, yellow fever, etc.), the introduction of which into the body is necessary to the production of the disease, or whether a combination of conditional causes (circumfusa), material and dynamia induce this without any such direct toxæmia, but rather as a dyscrasia or diathic affection. Having no time to dwell on this inquiry, I would merely suggest that in the whole range of nosology, the nearest analogy to diphtheria appears to be presented in erysipelas, in the relations of which—in origin, at once to the state of atmospheric, and to the state of the surface (and system at large) of the patient affected—we see much that is instructive and deserving of careful consideration.

It is probable that the infection of these two diseases may be found to have a similar rationale, and to be subject to the same laws of development and prevention.

*Treatment.*—Watson, Wood, Condie, C. F. Meigs and others, especially of the early writers, assert their confidence in the use of mercury with local applications. Some of the above and other writers commend the treatment with an emetic of antimony, and afterward in small or moderate doses. Bleeding from the arm in selected cases has been recommended by a few; leeching at the commencement of comparatively sthenic cases is advocated. Saline purgatives in the early stages are also approved by some. But the treatment upon which France and England have now most generally agreed, consists of the chlorate of potassa and tincture chloride iron, and the equally important employment of strong solutions of the nitrate of silver; grs. xxx to ℥j of water; or hydrochloric acid, equal parts, mixed with honey, or of Labarraque's chlorated solution ʒiss. to ʒx. water, fully and frequently applied to the throat, with the additional use of emetics in the croupal variety, and of stimulants in the most malignant type. I had been very much engaged in attending upon the sick soldiers of the late Confederate States Army, and returning to my office after night (located at that time in the village of Paris, Fauquier county, Va.), on the 17th day of November, 1862, I was immediately called to see Eveline, a fine, healthy, likely, colored woman, aged 26. I had seen her two days before in robust health; I found her having apparently undergone a remarkable loss of flesh for so short a time. She was tremulous, her countenance was anxious, haggard, her breathing laborious, stridulous, her body cold, her bowels constipated, mind clouded, pulse, 120, soft, weak, and she was very rapidly approaching to a comatose condition. The lymphatic glands of the throat were much swollen; gave evidence of having received a profound and sudden shock to her nervous system. The virus of the crotolus introduced into her system probably would not have produced a more prompt and decided shock to the great "nerve centres" of her system. And it is this "shock," in my opinion, that draws the grand distinction line of demarcation between diphtheria and all the other augnia. I am rather astonished to find no author speaks of the shock to the nervous system in diphtheria. It is always present, always perceivable; for, in the mildest form of the disease, the *little patients* will be found to "tremble." With some difficulty I prized open her mouth, pressed upon the base of the tongue with the handle of a spoon, and saw the tonsils, and the fauces covered with patches of a dirty, yellowish, white, leathery exudation, beneath which ulcers of a greenish, white color could be detected, and at the same time I saw my first case of diphtheria in this portion of the Piedmont region of Virginia. The palate and fauces were covered also with the same exudation beneath which the ulcerative process progressing could be perceived. I prescribed for this case (without any idea of any amelioration) according to the most urgent symptoms, and to prevent the evident tendency to death. I rubbed her chest and throat with equal parts of hartshorn and olive oil; cauterized her throat patiently and thoroughly with saturated solution, arg. nitris. Her throat to be mopped out aftarward every three hours with the following mixture: ℞. chlorate potassa, ʒii.; armenian bole, ʒij.; mel despumata,℥j.; aqua roseæ, ℥viiij.; mixed. To take every four hours, teaspoonful mur. tinct. ferri, and chlorate potassa, equal parts. Gave ten grs. pulv. jalap in strong coffee, without cream or sugar; to be followed in ten hours by sulph. quinine, grs. xxx; quinine to be repeated early next morning; aqua vitæ to be used freely; buttermilk, half water, at pleasure.

*September* 18, 12 *M.*—Is much relieved; bowels freely moved by the jalap; ulcers loosing their green dirty appearance; breathing improved; anxious expression of countenance passing off.

℞. Sulp. quinine, grs. x., morning, noon and night. Chlorate potassa and m. t. ferri to be continued, to gargle the throat with the solution chlor. potassa, etc. Nourishing fluid diet at will.

*September* 19, 1 *P. M.*—Is convalescing rapidly. ℞. Hot toddy four or five times in 24 hours; regimen continued with the internal remedies, in small doses; no relapse.

I was called on the 30th day of September, 1862, out of my immediate neighborhood to the family of Mr. N. L——, reached his house late at night, and found three of his children dead in the house, and five of the remaining members of his white family extremely ill with diphtheria, so much so, that I learned their cases were by the attending physician deemed hopeless. The mother and one of the living little boys were of the croupal variety, the three remaining cases were of the ulcerative variety and exhibited symptoms, closely resembling those of the colored woman Eveline. I made enquiry for the attending physician, learned he was "up-stairs," had retired, disheartened, broken down, and had left the request that "I should take charge of all the sick without any reference to him." I learned from the aunt, a most intelligent young lady, that she was "mopping out" the patients' throats every hour with a sturated solution of argentic nitratis, and was giving them a dose of sulphate of quinine, containing about one grain per dose; they had been vomited and purged freely; dismay and dispair was plainly and indelibly stamped upon the features of the mother and these sweet little children. I peremptorily ordered the mopping stopped. I gave them all, according to age, enough morphia to insure a few hours sleep, and to allay all the local and general irritation I could, which I considered had been increased, and "kept up" by the frequency, of the application of the strong solution of caustic. In its place I substituted the following wash for the throat: ℞. Chlorate potassa, ʒii.; aqua rosæ, ℥viii.; syrup quince seed, ℥ii.; sulp. morphia, grs. iv. Mixed. Apply to the throat as a wash or gargle every four or five hours. Tinct. iron and chlorate potassa to be taken in full doses, morning, noon and night, with 20 grs. sulp. quinine every fourth hour if awake, according to age, the 20 grains being issued as the standard adult dose. French brandy, old apple brandy or whisky to be given pro re nata. Rub the lymphatic glands, throat and chest with the following liniment: ℞. aqua ammonia, oleum oreganum, a a, ℥iv.; tinct. lyttae ℥ss. Mixed. Rub well night and morning, nourishing liquid diet as much as they will take without being forced upon them. Just about the break of day, when I thought I was through, and just about to request to be allowed to lie down, Mr. L. approached me, and said: "Doctor, we have been in such a state of excitement and confusion that I have had no time to see after my blacks, my servant woman you just saw says some of them are sick. Wont you go down to the cabins and prescribe for them? Accordingly, I went down, carefully and patiently, examining the throats of the blacks, old and young. In this large lot of negroes, I found nineteen serious cases of diphtheria. There were several other cases amongst them where the "shock" to the sensorium was so slight that I only thought it necessary to prescribe a gargle of chloride of sodium, and the bowels opened, if necessary, by the pulv. jalapæ in strong coffee. The remaining nineteen cases, I cauterized their throats once thoroughly (and only once) with a saturated solution of nitrate of silver; opened their bowels (when necessary) with pulv. jalap in coffee. After the cauterization the throats to be mopped with the following solution three times in the twenty-four hours: ℞. chlorate potassa, pulv. gum arabic, loaf sugar, a a, ʒij.; aqua rosæ, ℥viii. Mixed or used as a gargle. Chest, throat and glands to be rubbed with soluble liniment, and in some cases the throat and glands were ordered painted over with tinct. iodine fort. twice a day, then every four hours. To take chlorate potassa and m. t. iron and quinine in free doses, or Labarraque's chloriated solution, as preferred, with a free allowance of apple brandy or whisky, and a nourishing liquid diet. This constituted the greater part of the treatment adopted in these cases. I remained three days and nights at this house, and when I left I had the great satisfaction of knowing that without a single exception they were canvalescing rapidly. There were no subsequent relapses, and no deaths after I took charge of these sick. In the space of ten days, there was hardly a farm-house in my whole section that could not furnish several cases of diphtheria. It prevailed in every variety and form, although the ulcerative variety was the most common form to be met with, I found it to be decidedly asthenic in its character, and large doses of quinine and the stimulants and sustaining remedies were better borne in this "epidemic" and in the chronic diarrhœa of the war, than in any other diseases I have ever encountered. I repeatedly gave in this epidemic from thirty to sixty grains sulph. quinine, or half a pint of strong brandy, and believe I often saved the lives of my patients by so doing. The more profound and sudden the "shock" to the great "nerve centres," "paripassu," the larger the dose of stimulants required. It struck down the robust, in apparent good health, with all the suddenness and violence of a toxicological poison. Its depressing influence on the heart's action was equally profound and sudden. It required for its successful treatment to be met early and promptly with enormous doses of quinine, ammonia, whisky and other well-known stimulants. I gave repeatedly from thirty to sixty grains sulph. quinine with the happiest effects. Apple brandy or whisky could be drank with the same impunity as water.

In this epidemic of 1862, as it prevailed in this section of the Piedmont region, nothing but the most powerful stimulants, decided sedatives, and proper sustaining remedies, were at all admissible in its treatment. A man had as well attempt to put out the flames of a house on fire, as to attempt to stay the devastating progress of that terrible epidemic with his finely-constructed atomizers, and their pleasant and inefficient "sprays" of titania, etc. I would consider calomel hurtful in each and every-sized dose. I can but look upon emetics (more especially of antimonia et potassa tartras) as contraindicated in the treatment of epidemic diphtheria. As long as the blood is sufficiently arterialized, there seems in the croupal variety to be no immediate danger; but so soon as the countenance shows a want of oxygenized blood (by turning bluish), the danger becomes imminent. I think all emetics tend to increase this deoxygenation of the blood, and are therefore, in every variety of diphtheria, inadmissible in its treatment. Moreover, I do not like this plan of "blowing hot" and "blowing cold" at the same time, in prescribing your remedies. If diphtheria is an asthenic disease, all depletents, relaxents and depressents are hurtful, and stimulants, sustaining remedies beneficial;—if sthenic, stimulants, sedatives and sustaining remedies hurtful, and depletents, relaxents (such emetics) and depressents of service, and become the remedies for its proper treatment. So I can see no reason for blending different classes of remedial agents together in the treatment of diphtheria. I would no more think of attempting to bleed a patient in diphtheria than I would a patient prostrated from the bite of a poisonous reptile.

I have never found as much benefit from the local application of tincture of iodine as I would expect to, from the high praise bestowed upon it by many intelligent physicians. I think, of all the local applications, nitrate of silver is the best—to be applied thoroughly once, but only once, and then followed by mild, detergent and demulcent gargles. The next best local application to the nitrate of silver is the proto-sulphate of iron; and then I should say comes the carbolic acid, valuable as a local application. There is one internal remedy, in addition to those enumerated by me (which I have never used) that I think would prove itself invaluable in the treatment of this disease. I allude to the hydrate of chloral.

It will be seen, by what I have before said, that I believe the origin of diphtheria is from a pathogenic poison, generated by as yet an indefinable atmospheric distemperature, as depressing in its influence upon the heart's action and vital energies as the virus of the rattlesnake proves itself to be when introduced into the system of the mammalia. Whilst a perfect antidote has been long since discovered for the poisonous virus of the snake, none as yet has been discovered for the specific poison producing the strange, yet terrible disease, nosologically known as diphtheria. This disease can count distinguished victims by the thousand—victims to its fearful ravages. The death of General Washington and the Empress Josephine are attributed to it; Washington's death incorrectly, I think. Upon careful reading of the case prepared by the attending physicians, Drs. Dick and Craig, and the consulting physician, Dr. Gustavus Broun, of Potobacco, Maryland, I am satisfied this great and good man died from a "sporadic attack of sthenic tonsillitis," or common "quinsey." Josephine died either of the croupons variety of diphtheria, or from membranous croup. Some of the German writers, converts to the theory of the cryptogamous origin of diphtheria, are very decided in their recommendation of the "inhalation of bromine" for the treatment of diphtheria. Others, again, say it is better to combine equal proportions of "bromide of potassium" with the bromine. In our own county a most distinguished physician and surgeon has presented a very valuable paper, full of successful calculations, showing the great value of the inhalation of bromine in the treatment of this disease. Whilst I am not (by any means) a convert to the cryptogamous theory of this disease, yet, from my limited experience with the "inhalation of bromine," I am disposed to think highly of the remedial powers of this agent. I attribute its virtues, however, to its acting as a stimulant, generally and locally, to the diseased parts, just as I have seen a cloth dipped in aqua ammonia, and applied to the throat and chest, of equal value in membranous croup and diphtheria; causing the patient to cough up the false membrane in both of these terrible diseases. In the last month I have seen six or seven cases in my own practice of diphtheria, all being of the croupal variety, and all yielding to the plan of treatment as sketched by me for the treatment of asthenic diphtheria. Some cases occurred called diphtheria, which I placed to the credit of epidemic tonsillitis. I was called a few days ago in consultation to see a handsome little boy, six years of age. His physician was treating him with bilious purgatives, and with chlorate potash and quinine in minute doses, and vomiting him with ipecac, and applying a solution of argenti nitratis to the throat. He remarked to me that he thought him better since the last vomit had been given. He was quiet, composed, rational, and from his general appearance, one might have supposed there was but little the matter with the poor little fellow. His pulse was soft, compressible, slightly flagging, skin cool and moist, his throat apparently not very bad, but the exudation was extending downwards, and the countenance began to show a *bluish tint* in appearance. I gave my unfavorable prognosis, but advised free doses quinine, mur. tinct. ferri and whisky with soothing gargles and the "inhalation of bromine" in place of the further use of the nitrate of silver. The change was not made, and the little fellow succumbed within twenty-four hours from the time I saw him. I am persuaded the chief difficulty to the rational and systematic treatment of diphtheria lies in the fact that the physician does not, at the outset of the disease, thoroughly satisfy himself as to whether he has an "asthenic" or a "sthenic" case to deal with. If really "sthenic," use your lancet, your bilious purgatives, your emetics, your relaxants and your depletants,—and remedies generally, for here they are right and proper; but if the case is on the other hand, "asthenic," you must forego this class of remedies entirely, and rely upon stimulants, sedatives and the sustaining remedies. But do not mix your remedial agents; don't build your patients up a little by one remedy, whilst you pull them down a little with another. I wish it to be distinctly remembered that I am speaking of diphtheria as it occurred in the more mountainous regions of Piedmont Virginia; that I am ever mindful of the wonderful influence locality, etc., has upon epidemics. To prove this, I have only to quote from Dr. Honeyman on epidemic scarlatina. He is speaking of the dropsy following scarlatina, and says: "In this epidemic, digitalis, after proper preparation by the lancet, and sometimes aided by cupping and blistering, was just as much a specific as ever quinine was in my hands for intermittent fever." Strange to say, in an epidemic of scarlatina, in Piedmont Virginia (occurring at the same time) for the dropsy, following scarlatina, I found tonic doses of quinine never fail to act as *the* specific, and bleeding and digitalis would assuredly have proved fatal. As I have before said, there is an epidemic disease called "tonsillitis," sporadic cases of which do often take on a sthenic type. Membranous croup is also sthenic in its character. Nor am I prepared to deny that from some extraneous circumstances the serous membranes may not become involved in diphtheria as metastasis to the arachnoid of the brain; for instance, when the antiphlogistic remedies would undoubtedly be demanded.

But, nevertheless, from my experience, from analogy, and from all other sources of information accessible to me, I must say, if a physician recommends depletants in diphtheria, I simply doubt the correctness of his diagnosis, and believe he has confounded a case of membranous croup (it may be wearing some of the habiliments of diphtheria), with genuine diphtheria, which is always and everywhere an "asthenic" disease of the worst decided type, whose effect is a profound "shock," given as a sudden blow to the "great nerve centres," and whose secondary effect is as a "blood poisoner." I have seen the strong and manly heart cease to beat forever under the depressing influence of this subtle pathogenic poison, as suddenly as I ever saw it succumb to the deadly virus of poisonous reptilian. In a recent essay on diphtheria, the author draws a strongly-colored picture of the cruelty inflicted by the ignorant practitioner upon the poor, dear babies in his section. Whilst I have and do now accord general merit to the essay, I should nevertheless feel that I had done injustice to the noble profession of which I am an humble member, were I not to enter my solemn protest against such passages as the following. He says: "I have seen so much pain upon the young child, I have seen so much suffering inflicted and so much danger ensue by ramming a sponge or mop down the sensitive throat of a struggling baby, that I am sure I shall be throwing my influence in the scale of humanity by counseling entire abstinence from local treatment in the infant. Who has not observed the piteous and hopeless gaze of horror the young child fixes on the relentless knight of the probang, who approaches to repeat the suffocation of mopping? If the persecuted children could only speak, what a cry would go up to the merciless doctor. Let us die in peace. And let us not push a rude probang down the throat, and may be into the larynx, and through the vocal cords, possibly, in the vain hope of *punching* off and ramming home the pseudo-membrane, which is obstructing the ways of deglutition and respiration. And this leads us to the immitable caustic, for, of course, no doctor would think of ramming a sponge down a child's throat, whether into the pharynx or larynx (and who can tell which it goes into?), without dipping it first into a saturated solution of nitrate of silver." It is with genuine pleasure that I have to inform the medical profession of my own and sister States, that up here in Piedmont Virginia, where the sun shines brightly, and the sky is blue; where the water ripples down the hills and valleys in concert with the birds caroling their sweet songs upon each bough and tree, or speck of green foliage; where the young lambs frisk and gambol, and the cattle low in the velvety, emerald green fields, and the little child prattles gaily on the doctor's knee; where we have a little common sense and some discriminating judgment, these terrible blunders and horrid cruelties are unpracticed and unknown. It is at least presumable that our author never witnessed (as I have more than once) the late distinguished Dr. Horace Green catheterize the lungs for tuberculosis. But let us give a little authority as we go along. This plan of topical meditation to which M. Troussau alludes in his communication, was first brought before the profession of France in a paper presented by M. Troussau to the Academy of Medicine of Paris, in one of the sittings of that body in 1857, and which was entitled "An Easy Method of Entering the Air-Passages, in order to Catheterize them, or to Extract False Membrane; to Dilate the Glottis; to Introduce Substances used in the Treatment of Croup, either in the form of Liquid or Powder." This operation of catheterism of the larynx, says Professor Trousseau, is considered a very good means of taking the place of tracheotomy, and at all events should be tried before practicing that operation. On the reading of the paper of Loiscan before the Academy, M. Trousseau and Blanch were appointed a commission to report on the same, and their report made at a subsequent meeting was highly favorable to the plan of Loiscan, and was unanimously adopted by the Academy. The discussion that followed the report of the commission is of great interest. M. Depaul, alluding in the discussion to the declaration of the commission, that the process of catheterism of the larynx, as had been proposed, was very difficult of performance, said: "But I mention nothing is easier than this catheterism for those who have performed it a certain number of times." He did not "consider it equal in value to tracheotomy." "The operation of tracheotomy upon the larynx," said M. Piorry, "for diseases which are most frequently only secondary, are perhaps too much esteemed." In these cases the operation only shortens life, for they are useless in curing the primary disease. The operation of M. Loiscan, which at last is exempt from the dangers of tracheotomy, is preferable to the last. M. Velpeau declared that to M. Loiscan

"belonged the merit of having called attention to the subject." "Thanks to his memory," he continued, "we know that croup can be cured without operating for tracheotomy." That is a great deal. "I believe the operation recommended by M. Loiscan is a good one."

While diphtheritis is at the opening of the air-passages, it is curable, and M. Loiscan has ascertained that it is not difficult to carry medications into the larynx. The learned writer of the Gazette Medicale de Paris, in alluding to the discussion of the Academy, says of laryngeal cauterization: "As a therapeutical means it merits more serious attention. It is a powerful energetic means—the only one which up to this time has succeeded. When the disease is limited to the upper part of the air-passages, we cauterize, and *all* practitioners agree that this means is truly of great benefit. What is laryngeal cauterization other than carrying beyond the limits of ordinary cauterization in a remedy *recognized* as good, efficacious, not only against the *essence* of the disease itself, but also against the *pathological secretion ?* Laryngeal cauterization is then in this respect much superior to tracheotomy. Experience seems already to have confirmed these theoretical hopes; future experience will say much more of it." The beneficial results of cauterization in diphtheria is almost universally concurred in, and to-day as a topical application in the disease the nitrate argenti stands without a peer.

I claim no originality in the preparation of this paper, but have endeavored to make it an interesting compilation. I thought something of the kind was needed by the busy practitioner of medicine, and at some trouble I have tried to supply what I thought was demanded by the profession at the present time.

---

Subcutaneous Injections.—Dr. Constantin Paul recommends glycerine as a dissolvent for subcutaneous injections. He considers it to be far superior to water, alcohol, etc.; it is neutral, can be kept easily, and is, of all liquids, the one which approaches the nearest to the composition of subcutaneous cellular tissue. Glycerine is, indeed, almost a normal substance for cellulo-adipose tissues.—*Lancet.*

